# Obstetrics

**Published:** 1858-09

**Authors:** 


### OBSTETRICS.

1. On Menstruation during Pregnancy. *By Dr. Elsässer.*—This contribution to a disputed topic is founded upon fifty cases, extracted from the journal of the Stuttgart Lying-in Hospital—cases which are said to rest upon the most certain information. The subjects were fifteen primiparæ and thirty-six pluriparæ, who, with the exception of two women, (aged 36 and 41) were between 20 and 30 years of age. Of the fifty-one children born, thirty-four were boys and seventeen girls; thirty-six being mature and fifteen immature. The menstruation during pregnancy occurred in fifty women, in the following manner:—Once in eight, twice in ten, three times in twelve, four in five, five in six, eight in five, and nine in two. In thirteen cases the peculiarities of the rhythm of the discharge were inquired into, and the rhythm was found regular in four; in one it occurred at the sixth week; in three there were pauses between the epochs; in two the menstruation first appeared after the second month; in two after the fourth; and in one after the fifth month. In one case the menstruation first appeared in the middle of gestation, and henceforth came on every four weeks, lasting three or four days. The child, perceived but feebly at first, was strongly felt during the last four or five weeks. Hemorrhage occurred twice within a week before delivery, but a mature, living infant was born. Indications as to the amount of discharge were furnished in twenty-six cases, and in eighteen of them it was less than in the non-pregnant condition. The weight of the thirty-five mature infants varied from five pounds to nine pounds.

Dr. Elsässer observes that, although he is unable to state the proportion of cases in which menstruation occurs during pregnancy, it is by no means so exceptional an occurrence as supposed by some authors. It occurs more frequently in pluriparæ than in primiparæ; and it takes place much more frequently during the first half of pregnancy, and especially in the earlier months of this, than during the latter half. The amount of discharge, too, is smaller than in normal menstruation. The duration of the pregnancy was normal in more than two-thirds of these cases (thirty-six,) while in nearly one-third (fourteen) of the cases it was interrupted; in four during its first, and in ten during its latter half. As regards the development of the child, which by some authors has been supposed to be impeded by the occurrence of menstruation, this was found to be normal, or more than normal, in three-fourths of the cases.—*Monatschrift für Geburtskünde, band* lxxiii. pp. 401–408.—*Med. Times and Gazette,* April 24, 1858.

2. Observations on the Beneficial Effects of Pepsin in the Obstinate Vomiting of Pregnancy. *By Dr. L. Gros.*—In a great majority of cases the vomiting of pregnancy may safely be left to the influence of time; but there are some cases in which females are scarcely able to retain in their digestive system a sufficient amount of nourishment to support their existence, and are therefore reduced to the last degree of emaciation. In some, also, the shocks occasioned by this obstinate and repeated vomiting become the source of abortions, which might have been prevented by moderating the activity of the morbid phenomenon. A very remarkable case was related in 1856, by M. Teissier, Professor of Clinical

Medicine at Lyons, showing the immediately beneficial effects of a dose of pepsin in a case of vomiting during pregnancy. In this case the symptoms resisted all the ordinary methods which were employed, and the patient was unable to retain in her stomach any substance whatever. Under these circumstances, the patient was brought to M. Teissier, who found her in the following condition:—The vomiting had continued for two months, and she was at the end of the fourth month of her pregnancy; she presented the appearance of a skeleton, having the aspect and the cough of a phthisical subject; the pulse was 140, and M. Teissier thought at first that the case was one of pulmonary tubercle. Finding that all treatment had been hitherto inefficacious, and that the lady was fast actually dying of inanition, he was seriously meditating upon the propriety of inducing abortion as a means of saving her life; but as a last resource, before operating, he determined to employ pepsin. He accordingly prescribed one gramme, to be divided into two doses, and taken every day in a spoonful of broth. At the very first dose the broth was retained, and from that moment the vomiting never returned. On the third day the lady ate some chicken, and then some beefsteak. The treatment was continued in the same manner for three weeks, and at the end of that time the cure was complete; the emaciation was replaced by *embonpoint*, the fever and the cough ceased with the vomiting, and at the end of the nine months the lady was safely delivered.

Dr. Gros then relates six other cases in which the pepsin was employed with the same success, and he thinks himself warranted in concluding that pepsin undoubtedly produces good effects in the vomiting which attends pregnancy. He explains the results by supposing that, although in the first instance the vomiting is due only to the sympathy existing between the uterus and the stomach, yet subsequently the stomach itself becomes affected, as is proved by the fact that in the beginning of pregnancy the vomiting occurs only in the morning or the evening; but in aggravated cases it supervenes at every meal, and all alimentary matters are rejected. In such cases, therefore, when the stomach has taken on a morbid habit, and exhibits an alteration of secretion, the pepsin appears to be really indicated; although, in a merely sympathetic action between the uterus and stomach, it would be difficult to explain the efficacy of its action.—*Bulletin Général de Thérapeutique*, Feb. 15, 1858.—*British and Foreign Med.-Chir. Review*, July, 1858.

3. ON THE CARBONIC ACID GAS DOUCHE IN UTERINE DISEASE. By *M. Charles Bernard.*—Dr. Bernard reports very favorably of some trials he has made of Dr. Simpson's treatment of painful affections of the uterus by injections of carbonic acid gas. Of his successful cases he relates eight of the most marked, four being examples of advanced and very painful cancer, and four examples of engorgement or painful congestion of the cervix. The gas has been, however, employed in other cases in which no amelioration has attended its use, an aggravation of suffering being produced in one of them.

In all the patients, besides the local anæsthetic effect, more or less of general accidents were observed, these being usually very slight, but in one case so considerable as to compel the discontinuance of the remedy. They consisted in cephalalgia, giddiness, weakness, or confusion of vision, nausea, general lassitude, and more or less complete somnolence. After the third or fourth injection the patient complains of cephalalgia, and especially of somnolence, these increasing day by day, but usually soon passing off, except a slight abiding headache. In the case in which these symptoms persisted, they entirely resembled those of incomplete asphyxia from carbonic acid gas. These symptoms are, no doubt, due to the absorption of the gas. The general conclusions, then, are—1. Injections of carbonic acid gas constitute a powerful anæsthetic, and cause a rapid diminution of pain in an engorged or cancerous state of the cervix uteri. 2. In one case they seemed to expedite the resolution of a simple engorgement, and in another, to diminish a cancerous ulceration. 3. But they sometimes give rise to general disturbances, which, scarcely noticeable in open cancer, are more or less marked in simple engorgement.—*Archives Gén.* tom. x., pp. 529–548.—*Medical Times and Gazette*, April 10, 1858.

4. LAUDANUM DRESSINGS IN PAINFUL AFFECTIONS OF THE UTERUS. By *M. Aran.* —M. Aran, in this paper, describes a means of locally applying laudanum, that he has found useful in several hundred affections of the uterus. The object being to retain this substance in contact with the os uteri and upper part of the vagina, a magma is produced by means of an inert powder. The cervix being exposed by means of the speculum, from thirty to fifty (and sometimes more) drops of laudanum are allowed to flow to the bottom of the instrument. After bringing the fluid in contact with the surface by alternately opening and closing the valves of the speculum, a few drachms of starch are placed at the bottom of the instrument by means of a spoon or spatula, so that the laudanum may become absorbed by this, and, this taking place very speedily, the speculum is then withdrawn. While this is doing the starch is kept in the vagina by charpie or cotton, which, indeed, may be left at the entrance of the vulva, if the size of this leads to the fear that the substance will fall out when the patient stands erect. Absorption takes place slowly, one hour, and sometimes three or four hours, being required before the first assuaging effect is perceived. No ill effects, whether from excess of narcosis or disturbance of digestion, have been observed. The application may be repeated every other day, or even every day, the patient in the interval well washing out the previously applied magma.

This dressing is applicable to those affections of the uterus and genital organs which, after active inflammation is subdued, still manifest a painful hyperæsthesia. But it is especially useful in the hyperæsthesia which sometimes accompanies uterine deviations, or the morbid adhesions contracted by the uterus with the other pelvic organs. In these cases of chronic cellulitis, as also in chronic inflammation of the ovary and the tube, it does good service by allaying morbid sensibility. In such cases a precise indication may be often sought for in vain, nothing indicating inflammation or congestion, or these having been subdued by local bleeding. A few laudanum dressings quickly bring relief, and that not for some days only, but for entire months. "I have found another morbid occurrence remarkably modified by these dressings, viz., a special condition of the entire uterus, frequently met with in women of a certain age, and which, perhaps, is in some cases connected with the formation of small fibrous tumors. In these patients the finger and the speculum fail to detect any sign of inflammation or congestion; and yet so excessive is the morbid sensibility, that a false step or violent shock excites sensibility on every side; in other words, it is a most complete state of hysteralgia; a few laudanum dressings soon allay this sensibility and pain, the patient exchanging in a few days a state of extreme suffering and uneasiness for one of remarkable comfort." M. Aran has made but little use of this means in ulcerated cancer, fearing hemorrhage from the employment of the speculum; but in non-ulcerated cancer and epithelioma and in fibrous tumors, this means has afforded more marked relief than any other he has tried. It does not cure these patients; but to afford them relief is to do much.—*Bull. de Thérap.* tom. liii., p. 481.—*Medical Times and Gazette,* April 3, 1858.

